# Retraction: CircSETD3 (Hsa_circ_0000567) suppresses Hepatoblastoma pathogenesis via targeting the mir-423-3p/Bcl-2-interacting mediator of cell death axis

**DOI:** 10.3389/fgene.2024.1521304

**Published:** 2024-11-13

**Authors:** Frontiers Editorial Office

The publisher retracts the 29 September 2021 article cited above.

Following publication, concerns were raised regarding the integrity of the images in the published figures. The authors failed to provide the raw data or a satisfactory explanation during the investigation, which was conducted in accordance with Frontiers’ policies. Given the concerns about the validity of the data, and the lack of raw data, the editors no longer have confidence in the findings presented in the article.

This retraction was approved by the Chief Editors of Frontiers in Genetics and the Chief Executive Editor of Frontiers. The authors received a communication regarding the retraction and had a chance to respond. This communication has been recorded by the publisher.

Frontiers would like to thank the concerned readers who contacted us regarding the published article.

